# Discharge patterning in rat olfactory bulb mitral cells in vivo

**DOI:** 10.14814/phy2.12021

**Published:** 2014-10-03

**Authors:** Gareth Leng, Hirofumi Hashimoto, Chiharu Tsuji, Nancy Sabatier, Mike Ludwig

**Affiliations:** 1 Centre for Integrative Physiology, University of Edinburgh, Edinburgh, UK

**Keywords:** Mitral cells, olfactory bulb

## Abstract

Here we present a detailed statistical analysis of the discharge characteristics of mitral cells of the main olfactory bulb of urethane‐anesthetized rats. Neurons were recorded from the mitral cell layer, and antidromically identified by stimuli applied to the lateral olfactory tract. All mitral cells displayed repeated, prolonged bursts of action potentials typically lasting >100 sec and separated by similarly long intervals; about half were completely silent between bursts. No such bursting was observed in nonmitral cells recorded in close proximity to mitral cells. Bursts were asynchronous among even adjacent mitral cells. The intraburst activity of most mitral cells showed strong entrainment to the spontaneous respiratory rhythm; similar entrainment was seen in some, but not all nonmitral cells. All mitral cells displayed a peak of excitability at ~25 msec after spikes, as reflected by a peak in the interspike interval distribution and in the corresponding hazard function. About half also showed a peak at about 6 msec, reflecting the common occurrence of doublet spikes. Nonmitral cells showed no such doublet spikes. Bursts typically increased in intensity over the first 20–30 sec of a burst, during which time doublets were rare or absent. After 20–30 sec (in cells that exhibited doublets), doublets occurred frequently for as long as the burst persisted, in trains of up to 10 doublets. The last doublet was followed by an extended relative refractory period the duration of which was independent of train length. In cells that were excited by application of a particular odor, responsiveness was apparently greater during silent periods between bursts than during bursts. Conversely in cells that were inhibited by a particular odor, responsiveness was only apparent when cells were active. Extensive raw (event timing) data from the cells, together with details of those analyses, are provided as supplementary material, freely available for secondary use by others.

## Abstract (precis)

Here we present a detailed statistical analysis of the discharge characteristics of mitral cells of the main olfactory bulb of urethane‐anesthetized rats. Extensive raw (event timing) data from the cells, together with details of those analyses, are provided as supplementary material, freely available for secondary use by others.

## Introduction

The output of the main olfactory bulb is coded by the spiking activity of mitral and tufted cells: these neurons project to diverse brain sites via axons that leave the bulb in the lateral olfactory tract (LOT). The mitral cells have a primary dendrite that forms a highly branched tuft within a single glomerulus and several secondary dendrites which form dendrodendritic synapses on granule cells – axonless interneurons that are abundant in the olfactory bulb. These dendrites release glutamate in a calcium‐dependent manner that activates adjacent granule cells, and they in turn receive GABAergic inputs from the granule cells via dendrodendritic synapses (Aroniadou‐Anderjaska et al. 1999; Isaacson 2001). In addition, mitral cells may also detect glutamate that is released from their own dendrites or from those of neighboring mitral cells (Nicoll and Jahr 1982; Isaacson 1999; Carlson et al. 2000; Margrie et al. 2001; Salin et al. 2001). Synaptic transmission at these dendrodendritic synapses is characterized by its unusually slow kinetics (Mori and Takagi 1978; Nowycky et al. 1981; Jahr and Nicoll 1982) and by an unusual dependence on the activation of NMDA receptors (Isaacson and Strowbridge 1998; Schoppa et al. 1998); it has been suggested that these long‐lasting synaptic responses may be critical in the generation of oscillations and synchronous firing of neurons.

Yu et al. (1993) gave the first detailed description of the spontaneous firing patterns of mitral cells recorded in vivo from urethane‐anesthetized rats. In their experiments, units were recorded in a region characterized by the point at which the field potential evoked by stimulation of the LOT reversed in polarity, which is a convenient electrophysiological marker for the mitral cell body layer (Rall and Shepherd 1968), and within this region mitral cells were identified antidromically. In this they differed from many subsequent studies which have often relied only on the field potential or recording depth to identify cells as mitral cells. Most of the cells reported by Yu et al. (1993) showed prolonged bursts of activity, separated by similarly long periods during which activity was low or absent. Similar long bursts had previously been described in the miniature pig (Reinhardt et al. 1981), and similar bursts were later described in cats anesthetized with an initial dose of ketamine hydrochloride and maintained under anesthesia with intravenous urethane–chloralose (Motokizawa and Ogawa 1997). These authors also reported another feature of mitral cell activity for the first time – that the distribution of interspike intervals was often bimodal, with an early mode that reflects the frequent occurrence of spikes separated by very short intervals (<10 msec). That feature was also noted by Nica et al. (2010) in eight of the 29 mitral cells that they reported (recorded in the rat) – however, these authors did not describe long‐duration bursting, possibly because most of their recordings were relatively short in duration (median duration 400 sec).

These two distinctive features of mitral cell activity in vivo have received relatively little attention in the literature, in part, because neither of these features has been reported in vitro. The primary aim of the present study was to analyze the spontaneous firing characteristics of mitral cells, and in particular to study the structure of spike patterning with a view to inferring the underlying mechanisms, and their potential significance.

## Methods

All experiments were performed in accordance with Home Office (UK) regulations under appropriate project and personal licenses. Briefly, adult Sprague–Dawley rats (250–350 g) were anesthetized with urethane (ethyl carbamate; 25% weight/volume; 1.25 g/kg body weight i.p.). The rats were placed in a stereotaxic frame and a side‐by‐side bipolar‐stimulating electrode (Clark Electromedical Instruments, Kent, UK) was positioned on the LOT, below the piriform cortex, via a burr hole in the dorsal surface of the skull, 4 mm lateral and 2.2 mm anterior to bregma with the surface of the skull level between bregma and lambda (Paxinos and Watson 1986); the stimulating electrode was lowered until it was seen to bend slightly, indicating that it had reached the base of the brain, and then raised slightly. A recording electrode (glass micropipette filled with 0.9% NaCl, 20–40 MΩ) was lowered into the dorsally exposed, ipsilateral main olfactory bulb, at ~1 mm lateral to the midline and ~7 mm anterior to bregma. In initial experiments, the position of the stimulating electrode was verified by removing the brain after the experiment and fixing it in 4% paraformaldehyde. Sucrose was added to the fixative solution (as a cryoprotectant) along with potassium ferrocyanide and potassium ferricyanide (VWR, Lutterworth, UK). The brains were left in this solution for 2 days, a procedure that stains the tract yellow. Frozen brain sections (52‐*μ*m) were mounted on gelatine subbed slides and viewed using a binocular microscope.

Stimulation of the LOT consisted of a biphasic pulse (width 0.1 msec, 0.1–1 mA) peak‐to‐peak (S88 stimulator with stimulus isolation and current‐control units; Grass Medical Instruments, Warwick, RI) at 0.5 Hz. Evoked field potentials were used to indicate recording location. The mitral cell layer is identified by an isopotential and the granule cell layer by a positive phase to the second component of the evoked field potential.

### Antidromic identification

Classically, three tests are used to verify antidromic activation of single neurons: (1) spikes evoked by a stimulus should occur at a constant latency; (2) evoked spikes should be absent if the stimulus is preceded by a spontaneous spike within an interval that is the sum of the latency and the absolute refractory period (collision test); and (3) evoked spikes should faithfully follow short trains of stimuli at high frequency (100 Hz). We antidromically identified mitral cells by stimuli applied at a site where the LOT is a compact bundle, far from the main olfactory bulb (path length >5 mm) to maximize the latency of antidromic spikes, separating them well from the stimulus artifact. We used brief stimuli (0.1‐msec duration) to minimize the duration of the artifact, and reduced stimulus intensity to threshold to minimize its size and that of the evoked field potential. Cells recorded from the region of the mitral cell layer that we were unable to activate antidromically even with high intensities of stimulation, although other cells in the same region were antidromically activated, were classed as presumptive interneurons.

In some experiments, for double recordings, electrodes on separate manipulators were positioned with the tips ~100 *μ*m apart. In some other cases, two cells were recorded with the single recording electrode where the spike heights were sufficiently different to sort them reliably; these recordings were sorted using the LabSpike software generated by Bhumbra et al. (2004) and freely available from the CED website (http://www.ced.co.uk/upu.shtml).

Odors were applied via a polythene cannula (0.1 mm diameter) placed 3 mm in front of the nose. Cells were tested with a range of odors, including heptanol, hexanol, pentane, isopentane, methylpentan 2.4.diol, trizol, 2 propanol, 1‐butanol, cyclohexan, toluene, xylene, vanilla, lemon, and peppermint, using a 1‐mL syringe filled with odor‐saturated air; 0.1 mL air was ejected over 2 sec for each test (or in some cases at the same rate for longer periods); clean air had no effect on any cell tested in this way.

### Statistical analyses

For all cells, autocorrelation histograms were constructed over an 8‐sec window in bins of 10 msec, using CED spike 2 analysis software (Cambridge Electronic Design, Cambridge, UK). Interspike interval histograms were constructed in 1‐msec bins from at least 10 min of spontaneous discharge activity. From these, hazard functions (Sabatier et al. 2004; Sabatier and Leng 2008) were constructed in 1‐msec bins by the formula: (hazard in bin [*t*,* t *+**1]) = (number of intervals in bin [*t*,* t *+**1])/(number of intervals of length > *t*). Hazard functions represent interspike interval data as changes in excitability following a spike: for events that arise randomly, the envelope of the interevent interval distribution is a negative exponential, whereas the envelope of the corresponding hazard function is parallel to the time axis at a height that reflects the mean firing rate; spike‐dependent changes in excitability can thus be recognized by deviations from this. Conditional hazard functions were constructed by first sorting intervals according to the length of the preceding interspike interval, and then constructing hazard functions as above from intervals selected according to predetermined conditional rules. For some cells, poststimulus time histograms were constructed of the responses of cells to 100 sec of stimulation of the LOT at 1 Hz.

## Results

**Figure 1.**
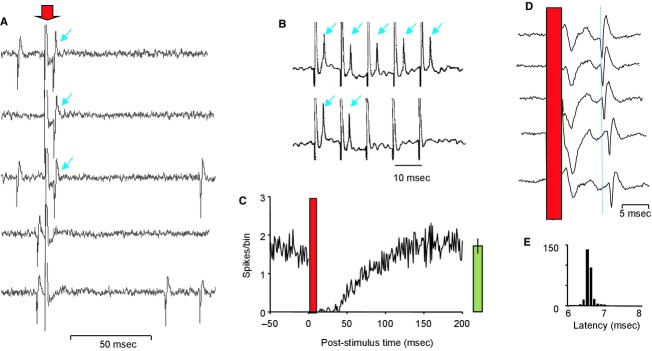
(A) Collision test for antidromic identification of mitral cells. Electrical stimuli (stimulus artifact marked with red arrow) applied to the lateral olfactory tract (LOT) evoke a constant latency spike (blue arrows) in the mitral cell unless the stimulus is immediately preceded by a spontaneous spike, as in the bottom two traces, when the antidromic spike is extinguished by collision. (B) Frequency following antidromically evoked spikes (arrows) follow stimuli applied at 100 Hz to the LOT with a constant latency. However, in the lower trace, the stimulus intensity has been *increased* by 50% and spikes no longer follow the stimuli faithfully, apparently because of increased lateral inhibition. (C) Average peristimulus time histogram for 29 mitral cells showing consistent suppression of activity for ~150 msec after LOT stimulation (at 1 Hz for 5 min). The stimulus artifact and antidromic spike events are in the period covered by the red bar. The bar on the right of the histogram shows the mean ± SE spikes/bin for the 29 mitral cells in the period 200–250 msec after stimulation. (D) Orthodromic excitation of interneurons in the olfactory bulb following LOT stimulation. Variable latency spikes (arrowed) following LOT stimulation (red bar). (E) Poststimulus time histogram of cell shown in D; orthodromic action potentials occur at a latency of between 6.5 and 6.9 msec.  **Data file (Figure S1) **EXCEL worksheet giving the peristimulus time histograms from 29 mitral cells, all from female rats, that contribute to the averaged data shown in C. The columns give spike counts in 1‐msec bins.

In initial experiments, 89 cells (from 50 female rats) recorded from the region of the mitral cell layer (as identified by the isopotential) were antidromically activated with a latency of 2.7 ± 0.2 (1.0–5.8) msec (mean ± SE (range)), at a threshold intensity of 0.3 ± 0.06 (0.1–0.5) mA (Fig. 1A). The ability of mitral cells to follow high‐frequency LOT stimulation was intensity‐ and frequency dependent, as first reported by Green et al. (1962). In the example in Figure 1B, stimulus pulses at an intensity close to threshold evoked a constant latency spike that was extinguished by collision with spontaneous spikes, but when the intensity was increased to twice threshold, the antidromically evoked spike failed 60% of the time. With a double‐pulse protocol, antidromic spikes followed pulses (at just suprathreshold intensity) faithfully when separated by 4 msec, but when pulses were separated by 7–24 msec, the spike after the second pulse failed occasionally, and when pulses were separated by 15 msec it failed consistently. At longer intervals (25–55 msec) the second spike was always present. Thus, LOT stimulation is followed by a delayed inhibition that is apparent between 7 and 25 msec after the stimulus and which can result in the failure of an antidromically evoked spike to invade the soma. The inhibition can be concluded to be delayed, because failure occurred with long but not short interpulse intervals, and it can be concluded that the inhibition is mediated transynaptically because in some cases we observed an inhibition longer than the expected interspike interval with high‐intensity stimulation (1.5–2 × threshold), but not at just suprathreshold intensities of stimulation.

After spontaneous spikes, mitral cells are generally relatively quiescent for only ~20 msec, but after antidromic invasion, as also originally reported by Green et al. (1962), mitral cells were inhibited by LOT stimulation (Fig. 1C) for 50 ± 9 (40–120) msec (*n* = 29 cells tested), indicating that LOT‐evoked inhibition is synaptically mediated. This involves lateral inhibition rather than recurrent inhibition alone because recurrent inhibition should be present after spontaneous spikes as well as after antidromic spikes. We therefore looked at the responses of nonmitral cells recorded from the region of the mitral cell layer, to see if the timing of their activation corresponded to the timing of inferred inhibition of mitral cells. These presumptive interneurons responded variably to LOT stimulation, but of 23 cells tested for their responses to 1 Hz stimulation, nine were strongly excited at a nearly constant short latency of 4–8 msec (intercell range; Fig. 1D and E). The other cells were either unresponsive or had late excitatory or inhibitory responses. Thus, in the region of the mitral cell layer, some interneurons displayed a nearly constant latency to LOT stimulation at latencies only slightly longer than the range for antidromically identified cells, at a timing that could account for their mediating the inhibitory effects of LOT stimulation upon mitral cells.

### Phasic bursting of mitral cells

Forty‐seven of the 89 mitral cells in female rats fired spontaneously with long bursts separated by long silent periods; these cells had an intraburst firing rate of 14.3 ± 1.1 (7.1–27.7) spikes/sec and an activity quotient (proportion of time active) of 50 ± 3 (21–77)%; thus typically they spent as much time silent as they did active. The mean burst length was 122 ± 10 (50–303) sec and the interburst time was 129 ± 11 (39–251) sec. The other 42 mitral cells also fired in long bursts, but were not silent between the bursts; these had an intraburst firing rate of 13.0 ± 1.5 (3.4–31.3) spikes/sec, a burst length of 103 ± 7 (27–292) sec, and an interburst time of 63 ± 8 (13–274) sec.

**Figure 2.**
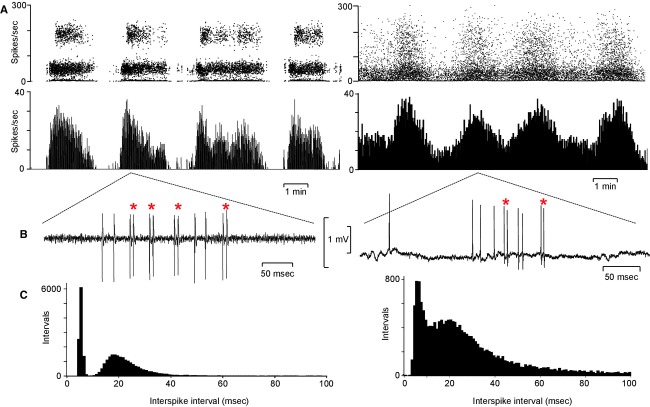
The phasic (slow bursting) activity of two mitral cells in the main olfactory bulb. (A) Instantaneous frequency plots; each point is the reciprocal of the preceding interspike interval. For the mitral cell shown on the left, the points fall in two bands: one band is spread around an instantaneous frequency of 200 spikes/sec, corresponding to interspike intervals of ~5 msec; the other band is spread around 40 spikes/sec, corresponding to interspike intervals of ~25 msec. The cell shown to the right is an example of a cell that was not completely silent between bursts, and for this cell there is a less clear separation between frequency modes. Below the instantaneous frequency plots are the corresponding ratemeter plots in 1‐sec bins. (B) Shows extracts of the raw signals showing doublet spikes (*). (C) Shows the corresponding interspike interval histograms (in 1‐msec bins), with modes at ~5 msec and at ~18 msec for both cells; the modes are clearly separated for the cell on the left.  **Data file (Figure S2)** Excel workbook giving the spike timing data for the two mitral cells extracts from which are shown in A, and the corresponding interspike interval distributions shown in C. Column A gives the recorded spike times (with a resolution of 0.1 msec) of 34,582 spikes in 3334 sec for one cell, column G gives the recorded spike times of 20,827 spikes in 1520 sec for the other cell. Columns B and H give the sequences of interspike intervals, and columns C and I give the reciprocals of these – the instantaneous firing rates as plotted in A. Columns E and K give the interspike interval distributions (in 1‐msec bins). The recorded spike times are relative to the first spike in the records.

In subsequent experiments in male rats we recorded from 94 antidromically identified mitral cells, all of which showed similar long bursting activity to that seen in female rats (Fig. 2). A main purpose of these experiments in male rats was to study the effects of vasopressin on the activity of the mitral cells, and those results have been published separately (Tobin et al. 2010). As briefly reported in that publication, these cells had a burst duration of 154 ± 7.2 sec, an interburst interval of 100.6 ± 8.4 sec, and an intraburst firing rate of 11 ± 0.5 spikes/sec (overall mean rate 6.4 ± 0.5 spikes/sec). In the present study, we analyzed 78 mitral cells from male rats, including many that were part of the set reported in Tobin et al. (2010). These cells were all recorded before any manipulations that might have had prolonged effects on mitral cell behavior; the analyzed recordings all included multiple long bursts, and were confirmed as free of artifacts or other disturbances (we retained the raw waveform records to allow us to verify this).

### Interspike interval distributions and hazard functions

**Figure 3.**
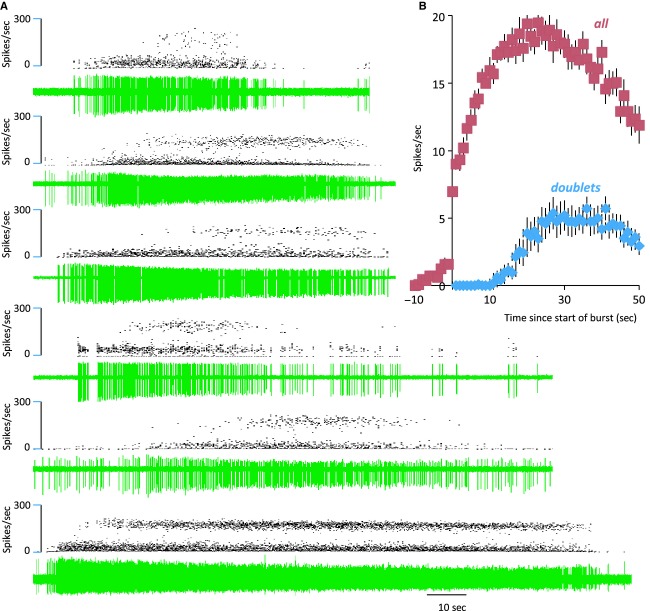
Evolution of bursts. (A) Examples of a single burst from each of five mitral cells; each trace shows the raw spike record in green below the corresponding instantaneous frequency record. Note that at the start of bursts there are no doublet spikes – these begin 20–30 sec after burst onset. Note also the reduction in spike height from the start of the burst. (B) Analysis of 17 successive bursts from a single mitral cell, mean firing rate (±SE in 1‐sec bins) relative to the start of bursts in red, and mean number of doublet spikes (intervals <10 msec).  **Data File (Figure S3)** Excel workbook (two pages) giving the data that contributes to B. The first page (“Burst profile”) includes 17 columns of data (B–R) each contain the spike counts in 1‐sec bins for a burst from the same mitral cell, aligned to the start of each burst (row 15) recognized as the first bin that contained at least four spikes that was followed by at least 20 bins all of which contained at least four spikes. The second page (“doublets”) shows the corresponding data giving just the number of doublets in each bin.

In both of the cells illustrated in Figure 2, the bursts (Fig. 2A) contain many examples of successive spikes occurring with very short interspike intervals (<10 msec; Fig. 2B). This is reflected in the bimodal distribution of interspike intervals (Fig. 2C); these occurred in trains of 2–12 spikes. Hereafter we call these short‐interval spikes “doublet spikes,” and spikes that occur after an interval >10 msec we call “single spikes.” In female rats, 51 of the 89 mitral cells had bimodal interspike interval distributions with modes at 5.2 ± 0.2 (2.5–8) msec and 20.3 ± 1.03 (12–38) msec; these we call “doublet cells.” Typically, doublet spikes occurred with a delay after the onset of the bursts (Fig. 3A); the average delay was 30.9 ± 2.5 (9–129) sec in such cells in female rats (Fig. 3B). The other mitral cells had a single mode at 23.2 ± 1.9 (5–56) msec, and these we call “nondoublet cells.” In all doublet cells, a doublet spike was usually followed by an interval >10 msec, but trains of two or three successive doublets were also common. In some cells, longer trains, of up to 10 successive doublets were observed.

**Figure 4.**
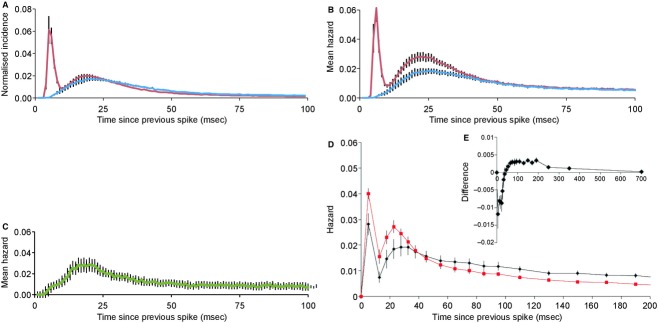
Intraburst discharge patterning of single olfactory bulb neurons and effects of doublet spikes on postspike excitability. Normalized interspike interval histograms (A) and corresponding mean hazard functions (B) of 48 mitral cells that fire doublet spikes (red); and 30 mitral cells with no doublet spikes (blue). Histograms were normalized to the total number of events, and averaged; the plots show mean ± SE. (C) Mean hazard function of 23 nonmitral cells (green). (D and E) For 23 bimodal mitral cells recorded from female rats, hazard functions were constructed for activity after either a long interspike interval (>10 msec), or a short interval (<10 msec). (D) Shows the mean hazard functions – the red symbols show the mean (SE) hazard after a long interval, and the black symbols show the mean hazard after a short interval. Comparing these, it is apparent that a short interval is followed by a suppression of excitability that results in a reduction in the hazard of firing for about 40 msec. The hazard at later times is greater after a short interval than after a long interval, consistent with the interpretation that doublet spikes tend to occur during sustained periods of depolarization. The inset (E) shows the mean (SE) difference in hazard functions, showing the apparent time course of the postdoublet effects on excitability.  **Data File (Figure S4)** Excel workbook giving, on the sheet labeled “D cells” the recorded event times of spikes recorded from each of the 48 doublet cells (mean 16,301 event times, mean duration 1850 sec), and on the sheet labeled “ND cells” the recorded event times of spikes recorded from each of the 30 nondoublet cells (mean 11,864 event times; mean duration 1865 sec). The sheets labeled “D isih” and “ND isih” give the corresponding interspike interval distributions (both raw and normalized), and the sheets labeled “D haz” and “ND haz” give the corresponding hazard functions. The sheet labeled “Example” gives an example of the construction of a hazard function from an interspike interval distribution.  **Data File (Figure S4A)** Excel workbook giving, on the sheet labeled “events” the recorded event times of spikes recorded from each of 24 nonmitral cells (presumptive interneurons). The sheets labeled “ISIH” and “Hazard” give the corresponding interspike interval distributions (both raw and normalized), and the corresponding hazard functions. The sheet labeled “Auto” gives the autocorrelation histograms: cells with conspicuous periodicity at ~0.8 Hz are headed by blue bars; those with little or no such periodicity are headed by red bars.  **Data File (Figure S4B)** Excel workbook giving, on the sheet labeled “Cells” the recorded spike times of 23 doublet cells recorded from female rats. The sheet labeled “Hazards” gives, for each of these cells, the calculated conditional hazards after long and short intervals, and the difference.

Of the 78 cells from male rats, 48 were doublet cells; they had interspike interval distributions with modes at 6.0 ± 0.1 (4–7) msec and at 22.3 ± 0.8 (14–39) msec (Fig. 4A, red); 30 cells were nondoublet cells with a single mode at 25.8 ± 1.1 (16–43) msec (Fig. 4A, blue; no significant differences to values in female rats). These interspike interval distributions were converted to hazard functions. The hazard functions of doublet cells had modes at 6.2 ± 0.13 (5–8) msec and 26.5 ± 0.99 (16–41) msec (Fig. 4B, red); those of nondoublet cells were unimodal with a mode at 28.8 ± 1.4 (13–41) msec (Fig. 4B, blue). Thus, doublet cells and nondoublet cell groups fire far from randomly within bursts, and show a peak of postspike excitability at ~27 msec corresponding to a preferred frequency of ~37 Hz. In this, these cells are unlike, for example: oxytocin cells in the hypothalamus, which, apart from a long relative refractory period, fire apparently randomly; vasopressin cells, which have a peak of excitability at ~40 msec corresponding to the peak of a depolarizing afterpotential (Sabatier et al. 2004); and all reported subtypes of neurons in the ventromedial nucleus of the hypothalamus (Sabatier and Leng 2008). In no mitral cell did we find a harmonic distribution of modes with a fundamental frequency corresponding to the ~27 msec mode; this suggests that there is no rhythmic forcing input to these cells that might explain a modal interspike interval at ~27 msec.

The mean firing rate of the doublet cells was 8.7 ± 0.67 (2.5–22.8) spikes/sec, that of nondoublet cells was significantly lower, at 6.6 ± 1.2 (1.3–15.3) spikes/sec (*P* < 0.05, *t* test). Comparing the two groups (Fig. 4B), the hazard functions after 50 msec are almost perfectly superimposable, but doublet cells not only have the additional early peak at ~6 msec but also have a higher hazard at ~27 msec.

We also constructed interspike interval distributions and hazard functions for 24 presumptive interneurons recorded in the region of the mitral cell layer. These all fired continuously at 10.2 ± 1.6 (3.3–22.4) spikes/sec. All had unimodal interspike interval distributions, but with a wide range of modes (mean 32 ± 5.6 [6–134] msec). The average hazard function had a maximum at 20 msec (Fig. 4C).

### Conditional hazard analysis

We asked how the excitability of mitral cells is affected by the immediate past history of spiking, by constructing *conditional hazard functions*. In particular, we asked how excitability changes after doublet spikes, by comparing hazard functions following doublet spikes with those following single spikes (Fig. 4D and E). Overall, a doublet spike is more likely to occur after a single spike than after a doublet (Fig. 4D), and from the difference in hazard functions (Fig. 4E) it appears that doublet spikes are followed by a greater activity‐dependent inhibition, lasting for ~50 msec.

**Figure 5.**
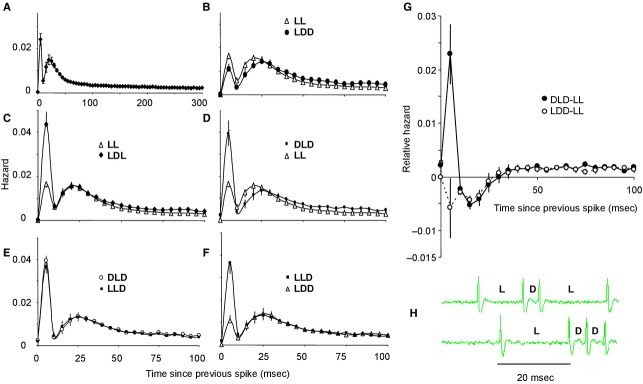
Conditional hazard analyses. (A–F) Analysis of 18 mitral cells, recorded from male rats, which fired with frequent doublet spikes. (A) Shows the mean (SE) hazard function; the function is bimodal, reflecting the frequent doublet firing. (B–F) Show mean *conditional* hazards, (B) shows the conditional hazards reflecting excitability after either a sequence of two long intervals (LL) or a long interval followed by two short intervals (long‐doublet‐doublet, LDD). Excitability is reduced for about 50 msec after LDD compared to after LL. (C) A doublet is more likely to follow a sequence of long‐doublet‐long (LDL) than two long intervals (LL). (D) A doublet is more likely to follow a sequence of doublet‐long‐doublet (DLD) than two long intervals (LL). (E) A doublet is equally likely to follow a sequence of DLD as it is to follow a sequence of long‐long‐doublet (LLD). (F) A doublet is more likely to follow a sequence of LLD than LDD. (G) For these mitral cells, we calculated conditional hazard functions to show how excitability evolves after (a) two long intervals (each < 10 msec; LL), (b) a sequence of doublet, long interval, doublet (DLD), and (c) after a sequence of long interval, doublet, doublet (LDD). The solid circles show the mean (±SE) *difference* between the hazard after LL and that after DLD, and the open circles the mean difference between the hazard after LL and that after LDD. After the first doublet of a train (i.e., after DLD) there is an enhanced chance of another spike occurring within 10 msec; but if this does not occur, then there is a reduced excitability for about 25 msec, consistent with the interpretation that a doublet spike triggers a fast DAP and a slow AHP. After two doublets (i.e., after LDD) there is generally a lower chance of another spike occurring within 10 msec, and there seems to be no prolongation of the AHP. (H) Examples of sequences LDL and LDD.  **Data File (Figure S5)** Excel workbook giving, for 18 doublet cells recorded from male rats, the full hazard functions (sheet labeled “Full haz”); and the corresponding conditional hazards after DLD, LLD, LL, LDL, LDD, and DDD in the sheets with these names, and (on “DLD vs. LL”) the differences between hazards after DLD and LL.

This initial analysis did not distinguish between long intervals that arise early in a burst (when there are few doublets) and those that arise later in a burst, many of which separate doublets. We therefore performed an extended conditional hazard analysis of an independent sample of 18 doublet cells from male rats (Fig. 5). For each cell, we constructed hazard functions of all activity (Fig. 5A), and hazard functions conditional on five histories: (1) after two long intervals (LL; two successive intervals >10 msec; Fig. 5B and C); (2) after two long intervals succeeded by a doublet spike (long‐long‐doublet, LLD; Fig. 5E and F); (3) after the second of the first two doublets of a train (long‐doublet‐doublet, LDD; Fig. 5B and F); (4) after a doublet within a sequence of doublets (doublet‐long‐doublet, DLD; Fig. 5D and E); and (5) after a long interval that followed an isolated doublet (long‐doublet‐long, LDL; Fig. 5C).

On average, the hazard function after DLD was almost exactly superimposable on that after LLD (Fig. 5E), but that after LDD showed a reduction in the early hazard (Fig. 5F), corresponding to a lowered probability of renewal of the doublet spike. A doublet is (generally) relatively unlikely to follow a sequence of two long intervals (LL), as most of these sequences occurred at the beginnings of bursts when doublets were rare or absent; conversely, a doublet is most likely to follow a sequence of long‐doublet‐long (LDL; Fig. 5C). After the first doublet of a train (i.e., after DLD or LLD) there is a relatively high chance of another spike occurring within 10 msec; but if this does not occur, then there is a *reduced* excitability for about 25 msec, as is apparent from the mean difference between the hazard after LL and that after DLD or LLD (Fig. 5G).

**Figure 6.**
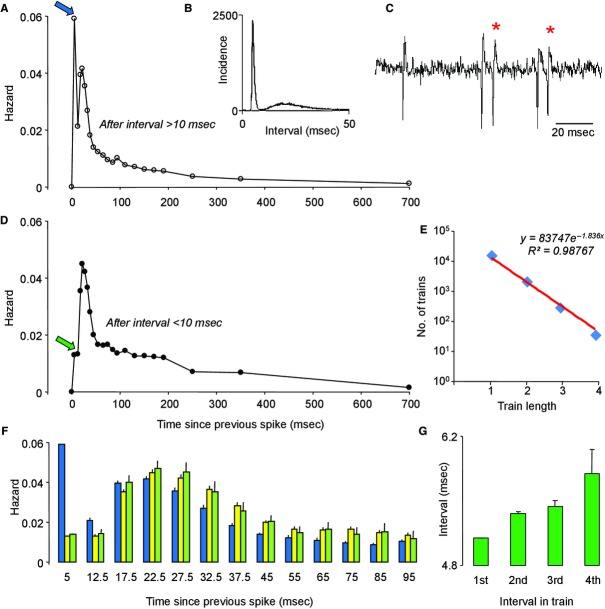
Conditional hazard analyses. (A) Conditional hazard function from a typical mitral cell, showing the hazard (per msec) of a spike occurring after a spike that followed an interval of >10 msec. Note the high probability (arrow) that the next spike will occur within 10 msec, giving a doublet spike. (B) Interspike interval distribution for this cell (in 0.1‐msec bins) showing the bimodal distribution characteristic of doublet cells. (C) Extract from the raw voltage trace showing examples of such doublet spikes (*). (D) By contrast with A, D shows the hazard of a spike occurring after a spike that followed an interval of <10 msec – that is after a doublet spike. The probability of another short interval occurring is very much lower (cf. open arrows in A and D). (E) Total number of instances (in a 1‐h recording) of trains of short intervals (<10 msec) of varying length. Note that the log of the number of instances is linear with train length. From this it may be deduced that the probability of renewal of the doublet spike is independent of the train length in this cell. (F) Hazards (with 95% confidence intervals) following long intervals (>10 msec, blue bars); one short interval (<10 msec, yellow bars) and two short intervals (green bars). Note that the hazard function after one short interval is identical to that after two. (G) Mean (SE) interspike interval for successive spikes in trains of short intervals. Note in this cell, the prolongation of interval during a train.  **Data File (Figure S6)** Excel workbook giving, in the sheet labeled “Hazard,” the conditional hazard functions plotted in A, D, and F. The sheet labeled “ISIH” gives the full interspike interval distribution for this cell, plotted in 0.1‐msec bins from 63,273 intervals. The spike timings of 2300 sec of activity (53,243 successive spikes) from this cell are given in the sheet labeled “Event times” (another ~10,000 intervals were included in the interspike interval distribution, from a separate recording sequence from this same cell). The sheet labeled “Trains” shows the detailed train analysis that gave rise to E and G.

After two doublets (i.e., after LDD) there was usually a lower chance of another spike occurring within 10 msec compared to DLD or LLD (Fig. 5F), but surprisingly, we found no additional prolongation of the postspike refractoriness. Moreover, not all cells showed a reduction in the probability of a doublet spike following two doublets. The cell analyzed in Figure 6 is a typical doublet cell, with a bimodal hazard function (Fig. 6A) and a bimodal distribution of interspike intervals (Fig. 6B) reflecting frequent doublet spikes arising at intervals of <10 msec (Fig. 6C). The hazard function following one doublet shows a marked reduction in the height of the first mode (Fig. 6D), showing that doublet spikes are more likely to occur after a long interval than immediately after another doublet. Nevertheless, this cell showed numerous trains of doublets, comprising up to eight spikes. Interestingly, the hazard following one doublet is indistinguishable from that following two successive doublets (Fig. 6F). Indeed, for this cell, the numbers of trains of different lengths was almost perfectly log linear with train length (Fig. 6E), suggesting that the probability of renewal of a doublet spike was independent of train length. In this cell, the interval between successive spikes in a train of doublets increased progressively during trains (Fig. 6G).

**Figure 7.**
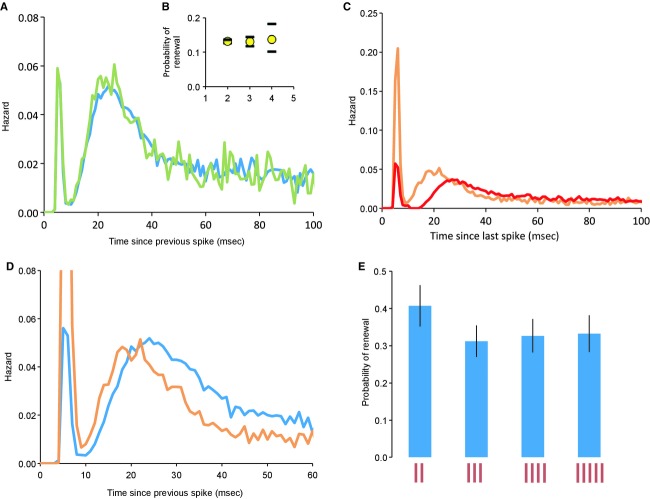
Hazard analysis of a single mitral cell. (A) Shows conditional hazard functions showing the hazard after the first short interval (<10 msec) of trains of short intervals (in blue), and after the second short interval of trains (green). Notice that the hazards closely superimpose. (B) Shows the renewal probability of doublets in this cell (bars show 95% confidence limits) after the first, second and third doublets of a train. (C) Shows the conditional hazard following two long intervals (orange) and the conditional hazard for doublets alone (red). Note that after a long interval, there is a high chance that the next spike will be a doublet, and if it is not a doublet the modal interval is ~20 msec. After a doublet spike, if another short interval does not follow immediately the next short interval is most likely to occur after an interval of ~30 msec. (D) Compares the hazard after two long intervals (orange) with that following the first doublet of a train (blue). Note that, after a doublet, excitability is reduced for about 20 msec. (E) Mean (SE) from 10 mitral cells that showed spike trains of up to eight spikes separated by short intervals (<10 msec). The bars show the probability of renewal given the past history – that is the probability that after one short interval (II), the next interval will be short, the next will also be short, etc. After two short intervals (III), the probability of renewal is lower, but remains stable thereafter.  **Data File (Figure S7)** Excel workbook giving, on the sheet labeled “Hazards,” the analysis of a single doublet cell illustrated in A–D. Column A gives the recorded spike times, column D–F gives intervals that follow two long intervals (D), that follow the first doublet of a train (E), and that follow the second doublet of a train (F). Columns I–N give the corresponding interspike interval distributions and hazard functions. Columns P–T give the distribution and hazard functions for doublets alone. The sheet labeled “Trains” gives, for each of 33 doublet cells, the total number of trains recorded, and the numbers of trains with at least 2, 3, 4 and 5 doublets. The sheet labeled “Renewals” gives the analysis shown in E of the 10 cells that showed frequent long trains.

We replicated this analysis in 10 doublet cells selected as showing frequent long trains of doublets. For the cell illustrated in Figure 7A, like that shown in Figure 6, the hazard function after the first doublet of a train was almost exactly superimposable on the hazard function after the second doublet, showing no additive effects on postspike excitability. The same was true for the hazard function after the third doublet of a train, hence the doublet renewal probability remained constant (Fig. 7B). For these 10 cells, we calculated the mean doublet renewal probabilities – on average, there was a slightly lower renewal probability after the second doublet of a train, but the renewal probabilities after the third and fourth doublets were similar to those after the second (Fig. 7E).

For the same cell as shown in Figure 7A and C compares the hazard function *for doublets alone* (i.e., the chance of getting a doublet spike as a function of time since the previous doublet), to the hazard function following two long intervals (LL). In this cell, whereas spikes generally arose with a modal interspike interval of ~20 msec, doublets arose with a modal interval of ~30 msec. This was not simply attributable to the reduced excitability following a doublet: Figure 7D compares the hazard after LL with that after the first doublet of a train, as expected (from Fig. 5) there is an extended refractoriness after a doublet, but this is not enough to account for the delayed mode of interdoublet intervals.

Thus, after a doublet spike, the duration of postspike hypoexcitability is longer than normal, and the subsequent peak of excitability is at about 30 msec. If a spike arises earlier than 25 msec, it is likely to be a single spike. However, if a spike arises later than this, it is highly likely to be a doublet spike. Thus, whereas spikes (in doublet cells) generally tend to recur at intervals of 25 msec, corresponding to a preferred frequency of ~40 Hz, doublets tend to recur at intervals of 30 msec, corresponding to a preferred frequency of ~30 Hz.

### Autocorrelations

**Figure 8.**
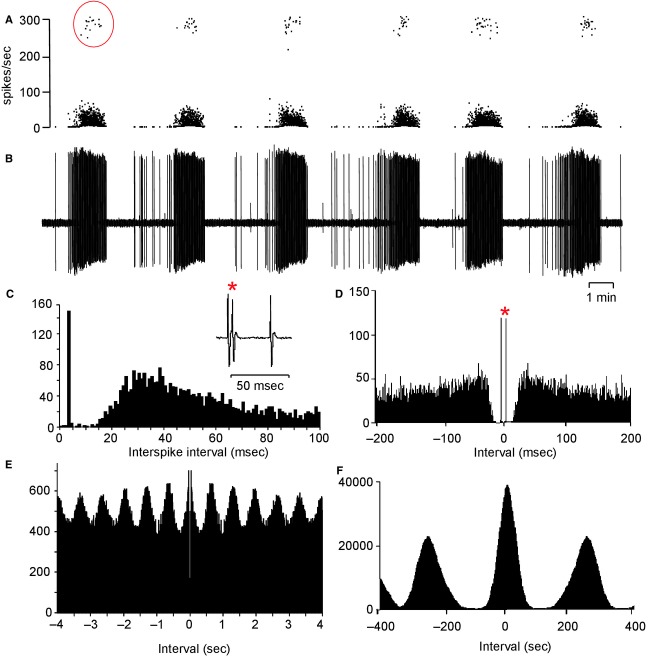
Autocorrelation analyses. (A) Instantaneous frequency record of a mitral cell, showing repeated long bursts which included instances of “doublet” spikes (examples circled). (B) Shows the raw voltage record corresponding to A. (C) Shows the interspike interval distribution of the cell in A, B, showing a bimodal distribution of intervals with the early mode corresponding to doublet spikes – an example of which is shown (*) in the inset. (D–F) Autocorrelation histograms constructed over different time scales. (D) In 1‐msec bins reveals the doublet spikes as large peaks at ±4 msec (*). (E) In 100‐msec bins, shows a respiratory rhythm with a period of ~800 msec. (F) In 1‐sec bins, shows the long periodicity of the slow bursting.  **Data File (Figure S8)** Excel workbook giving, on the sheet labeled “C–F,” the raw data for the histograms shown in Figure 10C–F. The sheets labeled “D cells” and “ND cells” give the autocorrelation histograms (in 10‐msec bins, over 8 sec) for 47 doublet cells and the 30 nondoublet cells for which the event timings are given in the Data file for Figure 5. Cells with conspicuous periodicity at ~0.8 Hz are headed by blue bars; those with little or no such periodicity are headed by red bars.

Eighty‐two of the 89 mitral cells recorded from female rats were analyzed with autocorrelation histograms (seven did not have a sufficiently long period of stable spontaneous activity; Fig. 8). On a timescale of 8‐sec (Fig. 8E), peaks were evident in the autocorrelation histograms of both doublet and nondoublet cells at times corresponding to rhythmic discharge at 1.5 Hz, consistent with a respiratory rhythm (the mean respiratory rate measured in a sample of eight rats was 89 ± 2 breaths/min), and the peaks were regular and well defined in most mitral cells (60/82). Autocorrelation histograms were constructed for all 78 mitral cells from male rats; a similar respiratory rhythm was marked in 36 of the 48 doublet cells and in 22 of the 30 nondoublet cells. Autocorrelation histograms were also constructed for the 24 nonmitral cells; only eight of these showed a marked respiratory rhythm.

### Dual recordings

To determine whether the phasic discharge pattern of mitral cells described was synchronous throughout the bulb, simultaneous recordings of two individual cells were made from six female rats. Only one of these six pairs of cells showed any correlation between bursts; for this pair, bursts in one cell consistently preceded those in the other by a mean of 26 sec. The other five pairs showed no apparent correlation in burst activity.

**Figure 9.**
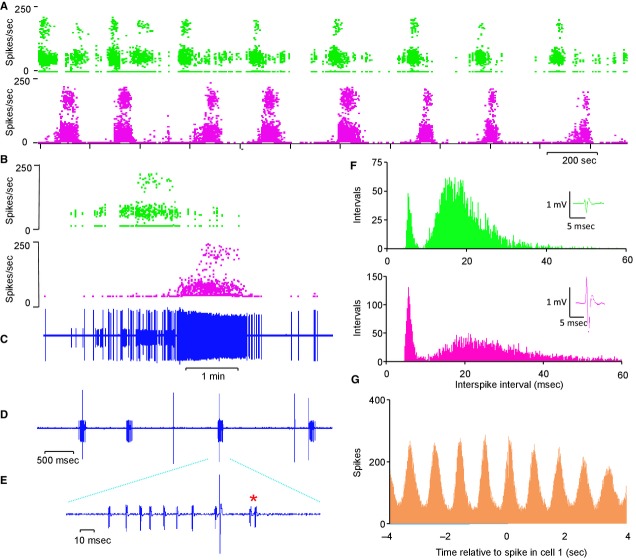
Simultaneous recording of two mitral cells recorded with a single electrode. (A) Instantaneous firing rates; the cells fired in long bursts that were asynchronous. Periods of doublet firing (instantaneous rates >100 spikes/sec) were mutually exclusive. (B) Expansion of a period covering one burst from each cell above the voltage record (C), which shows the large difference in spike amplitudes that made separation possible. (D) Shows a further expansion of the voltage trace to display the rhythmic intraburst activity locked to respiratory rhythm, and (E) shows a further expansion showing doublet firing (*) in the smaller cell. (F) Shows interspike interval distributions for the two cells. (G) Cross‐correlation of spike times; the correlation is wholly attributable to a common correlation with respiratory rhythm.  **Data File (Figure S9)** Excel workbook giving the histograms shown in F and G.

In four later experiments in male rats we could record a pair of antidromically identified mitral cells with a single electrode, where differences in spike heights allowed us to distinguish the cells clearly (Fig. 9). In each case, the bursts were asynchronous. The pair of cells shown in Figure 9A were both doublet cells (Fig. 9F), both of which showed a strong respiratory rhythm (Fig. 9D), but there was a consistent lag between the bursts in the adjacent cells, such that each of the cells fired in doublets only at times when the other did not (Fig. 9A). Cross‐correlations of their activities showed a common association with respiratory drive, but no evidence of direct interaction (Fig. 9G).

### Odor responses

**Figure 10.**
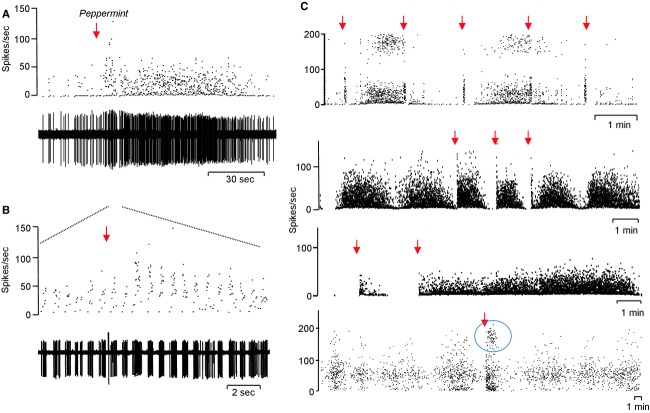
Examples of odor responses of mitral cells. (A) Prolonged response of a mitral cell to a 2‐sec exposure to peppermint odor (arrow): instantaneous frequency record above corresponding raw spike record. (B) Shows an expansion of part of (A) showing that activity is strongly modulated by respiratory rhythm. (C) Composite showing responses (instantaneous frequency records) of four other mitral cells to brief exposure to an odor (arrowed); responses are of variable duration within and between cells, but the initial response is consistent between applications within cells. Of the four cells, in only the last cell did exposure to the odor conspicuously evoke doublet firing (circled).

We tested each cell sequentially for their responses to a panel of odorants until an odor was found that elicited a clear response; we then studied how responses differed according to when, relative to the burst cycle, the stimulus was applied. Here, we report only on those cells where we were able to find clear and consistent responsiveness to a particular odor (Fig. 10). Individual mitral cells (*n* = 31) were identified as responding to odors, and these responses were, as far as we could tell, highly selective; two cells were activated by both toluene and xylene, but otherwise we found no cells activated by any other combinations (though most cells were tested with only a subset of odors). Most of the responses were excitations, but this may reflect a bias in that modest excitations were easier to reliably detect (when applied during silent periods) than modest inhibitions. Thus, when odors were applied during silent periods without effect, we did not routinely repeat those tests during periods of high activity, preferring to test a new odor instead. All responses of mitral cells were simple, comprising only excitation or inhibition – we observed no complex sequences of excitation and inhibition, though we did observe some complex responses in nonmitral cells recorded close to the mitral cells.

**Figure 11.**
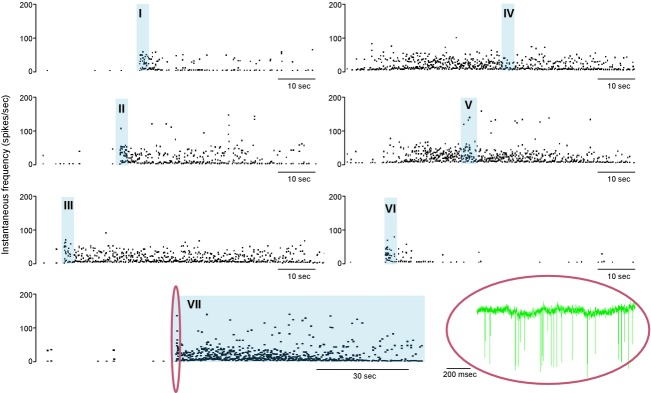
Responses of a mitral cell to hexanol (blue shaded bars). Applications I, II, and III were all applied during a silent period between bursts, and each triggered a prolonged burst of activity. Applications IV and V given during an active period elicited no clear response. Application VI given during a silent interval elicited a clear but short response. Application VII was a sustained application, and firing persisted throughout the period of application. The raw spike record corresponding to the region enclosed by the red oval is shown at bottom right.

When odors were applied during silent periods, the responses of mitral cells were very consistent during the odor application, but differed in that odor application often initiated long bursts of variable duration (Fig. 10). Figure 10A shows one example of a long burst triggered by brief exposure to peppermint odor applied during a quiescent period between bursts. In 10 sec after peppermint was applied, the respiratory‐driven activity intensified as expected (Fig. 10B), and as has been described extensively by others (e.g., Cang and Isaacson 2003); this odor‐driven activity was then followed by a long burst similar to spontaneous bursts. Burst triggering was erratic, both within cells and between cells. Four further examples are shown in Figure 10C; in the cell shown at the top, odor‐driven activation tended to stop bursts when given during a burst, but did not trigger bursts when given during silent intervals. By contrast, in the cell immediately below, odors repeatedly triggered long bursts. Below that, is a cell where odor application triggered bursts of variable duration. In these three cases, the odor application was excitatory but did not conspicuously induce doublet firing, the fourth cell shown at the bottom of Figure 10C is an exception in which doublets were specifically associated with odor application. In general, the likelihood of triggering a burst depended on the time elapsed since the end of the previous bursts, in that odors applied just after the end of a burst evoked only a brief excitation. Thus, in the cell illustrated in Figure 11, identical applications of the same odor all triggered bursts (of very differing durations) when given during silent periods, but when given during active periods had no clear effect.

**Figure 12.**
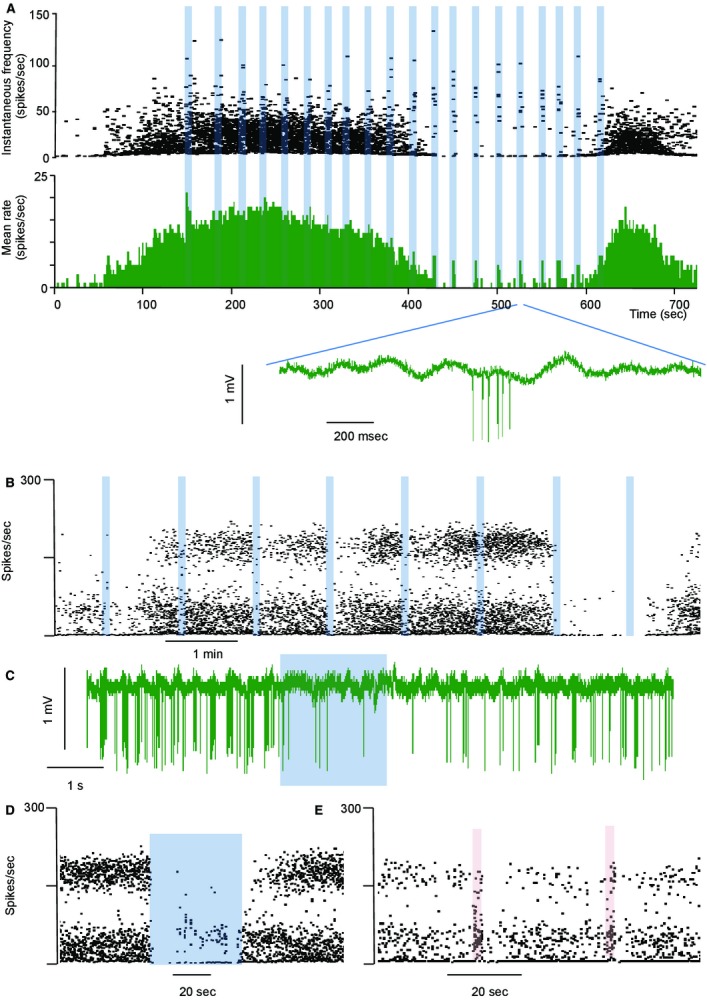
Responses of a mitral cell to repeated odor exposures (shaded bars). (A) The top record shows instantaneous frequency, below this in green is the corresponding rate record (in 1‐sec bins). Below this is an extract of the raw voltage trace. This cell responded consistently to hexanol application when silent; when active, responses were hard to discern. Instantaneous frequency plot of a mitral cell, showing (B), inhibition in response to heptanol (blue shaded bars). (C) Voltage record of a single inhibitory response to heptanol (blue bar). (D) Sustained inhibition of the same cell in response to heptanol – note that burst firing resumed immediately after the end of application. (E) Activation of the same cell by pentane (red bars), with postactivation inhibition.

Although excitatory responses were readily detected when applied during silent periods, responses during active periods were not always clear (Fig. 12A). Unsurprisingly, inhibitory responses were evident only when cells were active (Fig. 12B). While excitatory responses often triggered bursts, inhibitory responses seldom terminated bursts. Thus, the cell illustrated in Figure 12D showed sustained inhibition when odor was continuously applied for 40 sec during a burst, but the burst activity resumed promptly on termination of odor application. The same cell was activated by exposure to a different odorant (Fig. 12E).

## Discussion

The spontaneous discharge pattern of mitral cells in urethane‐anesthetized rats has three levels of patterned activity. (1) The most noticeable patterning comprises long periods of silence or relative quiescence between long bursts of high activity. (2) Within these long bursts, most mitral cells showed rhythmic activity at ~1.5 Hz which corresponds with the respiratory rate. (3) The third feature, exhibited by about 60% of mitral cells, is evident from their bimodal interspike interval distribution, with a short mode corresponding to spike intervals of <10 msec, and a second mode at about 25 msec. In most mitral cells that showed a bimodal interval distribution, bursts began with few or no doublet spikes, but as the mean intraburst frequency approached a maximum, trains of (usually 1–3 but sometimes up to 10) doublets occurred repeatedly until the burst ended.

### Bursting behavior

Long‐duration bursting has not been observed in mitral cells recorded in vitro*,* possibly simply because spontaneous firing rates are much lower in vitro than in vivo. However, it has been observed that mitral cells have an intrinsic membrane bistability; they can spontaneously oscillate between two membrane potentials separated by ~10 mV, and transitions between states can be triggered by changes in spike activity (Heyward et al. 2001). This suggests an obvious intrinsic process underlying the long bursts.

The spontaneous activity patterning of mitral cells does not reflect only intrinsic mechanisms; these patterns are also molded by network interactions within the bulb – by lateral inhibition and/or recurrent inhibition from interneurons. These influences are apparent both in the inhibitory effects of LOT stimulation, and strikingly in the ability of LOT stimulation to suppress antidromic invasion of mitral cells with an inferred delay of ~5 msec after antidromic invasion, which suggests a powerful inhibition of the soma itself, and they are consistent with short‐latency orthodromic activation of interneurons close to the mitral cell layer. In addition, glutamate released at dendrodendritic synapses is thought to have a long‐lasting depolarizing effect on the mitral cell of origin (Carlson et al. 2000; Christie and Westbrook 2006), and this may contribute to the activity‐dependent evolution of the prolonged bursts that, in this study in urethane‐anesthetized rats, were a universal, characteristic, and distinctive feature of mitral cells.

Heyward et al. (2001) showed that most mitral cells in vitro exhibit spontaneous alternations between an upstate – a membrane potential close to threshold for spike initiation – and a more hyperpolarized downstate. Transitions to the upstate could be triggered by brief depolarization, and this property appears to be intrinsic to the mitral cells. The upstate has the characteristics of a depolarized plateau potential, like that which supports bursting in several neuronal cell types. For this to explain bursting in mitral cells, it is necessary to envisage how the upstate might be regenerated by spike activity. The ability of brief odor application to trigger bursts in mitral cells suggests that bursting is indeed activity dependent, and activity‐dependent glutamate release from mitral cell dendrites provides a likely mechanism for this. However, while bursts could be readily triggered by brief activation, they were less likely to be interrupted by brief inhibitions, even though, as Heyward et al. (2001) described, transitions from the upstate to the downstate could be triggered by brief hyperpolarizations. Thus, if the bursts are indeed underpinned by an activity‐dependent plateau potential, this plateau potential seems to be relatively resistant to interruption by transient inhibition, and how this resistance arises is unclear – and how the bursts ultimately end also needs consideration.

Vasopressin cells of the rat hypothalamus also display long bursts separated by long silent intervals, and these bursts are sustained by a regenerative activity‐dependent mechanism, and can be triggered by brief stimuli applied toward the end of a silent period (Sabatier and Leng 2007). However, the same stimuli given early in the silent period may evoke only an immediate response or occasionally a truncated burst – apparently as we observed here. However, while it is easy to briefly interrupt bursts by brief inhibitory stimuli, only relatively prolonged inhibitions will terminate bursts. (In vasopressin cells, bursts are terminated by a slow and delayed activity‐dependent inhibition that imposes a very prolonged refractory period between bursts).

### Doublet spikes

In many mitral cells, as bursts progress, they display frequent “doublet spikes.” Several mechanisms can give rise to doublet spikes, but as the primary dendrites of mitral cells have high densities of sodium channels and action potentials propagate in these dendrites without attenuation (Bischofberger and Jonas 1997; Chen et al. 1997), an obvious mechanism is that doublet spikes reflect a short depolarizing afterpotential that reflects a depolarizing return current following dendritic propagation of the spike. In cells in which doublet spikes arise by this mechanism, the duration of the depolarizing afterpotential is proportional to the length of the dendritic path; if dendrites are truncated, the depolarization is too short lasting to exceed the absolute refractory period of the neuron, so no doublets can arise (Bekkers and Häusser 2007). This might explain why doublets are not seen in mitral cells recorded in slice preparations, in which many primary dendrites may be damaged or severed toward their distal ends.

Doublets generally are believed to be important for signaling because they are likely to produce facilitation of neurotransmitter release. The secondary dendrites of mitral cells also actively propagate action potentials, and these control lateral and recurrent inhibition (Margrie et al. 2001; Lowe 2002). Single action potentials attenuate as they propagate in secondary dendrites (Christie and Westbrook 2003), and dendritic glutamate release requires strong depolarization (Xiong and Chen 2002) and activation of high‐voltage gated calcium channels (Isaacson and Strowbridge 1998). Accordingly, the distal regions of these dendrites may not experience significant depolarization following single somatic action potentials, but may do after doublet spikes, which would thus be expected to be followed by enhanced recurrent inhibition. This seems a likely explanation of the extended refractoriness that characteristically follows doublet spikes. Recurrent inhibition is not the only possible explanation, but it seems more plausible than the alternative of an enhanced intrinsic afterhyperpolarization. We would expect that an intrinsic afterhyperpolarization would lead to a deterministic truncation of trains of doublets – and that the duration of refractoriness after a train of doublets would be extended as train length increased. Some mitral cells showed abundant doublets but very few trains of doublets, but others exhibited frequent longer trains, of up to 10 successive doublets, with an apparently random process governing train length. Interestingly, the hazard function following trains of doublets was consistently exactly superimposable on that which followed single doublets, indicating no prolongation of afterhyperpolarization and no potentiation of recurrent inhibition.

To explain the shape of the hazard functions observed in mitral cells, we must recognize that their spike activity is subject to an absolute refractory period of ~3 msec. Second, spikes are followed by a period of lower excitability, likely to reflect either a hyperpolarizing afterpotential or recurrent inhibition, and this is succeeded by a period of increased excitability, possibly reflecting dendritic glutamate spillover from spike‐dependent glutamate release at dendrites. Whatever the underlying mechanisms, this late excitation leads to a progressive increase in spike activity, generating a long burst. When the resting potential of the mitral cells is sufficiently depolarized through this activity‐dependent excitation, doublet spikes, occurring typically at intervals of 6–8 msec, begin to appear. These apparently arise as a result of a fast depolarizing afterpotential, possibly reflecting a dendritic return current that reaches spike threshold if the soma is already sufficiently depolarized, and they can occur in probabilistically regenerative trains of doublets of random length. These trains are followed by an extended period of relative inexcitability that apparently reflects recurrent inhibition, but which is independent of the length of the preceding train; we accordingly suggest that this inhibition arises stochastically as a result of doublet activity. The long bursts themselves appear to be terminated in an activity‐dependent manner. What causes their termination is not known; similarly long bursts in hypothalamic vasopressin cells are terminated by the actions of a dendritically released slow autocrine messenger (Brown et al. 2013), but we know of no evidence for a similar mechanism in mitral cells.

There have been many attempts to model the electrical activity of mitral cells, incorporating biophysical data on channel properties as inferred from experiments in vitro (see, e.g., Rubin and Cleland 2006; Arruda et al. 2013; Yu et al. 2013). Unfortunately, none of these models exhibit neither the long bursting behavior that here we report as a universal characteristic of rat mitral cells in vivo nor, with one partial exception, do they display the doublet spike behavior that is a conspicuous feature of about half of the mitral cells. The single exception that we are aware of is Bhalla and Bower's model (1993), in which spikes initiated at the soma by current clamp or antidromic stimuli may propagate dendritically to initiate a calcium spike in the glomerular tuft, and the resulting prolonged depolarization may initiate a second somatic spike. Interestingly, because doublet spikes in mitral cells have not generally been recognized as a common occurrence, Davison et al. (2003) concluded that the glomerular tuft in Bhalla and Bower's model was overexcitable, and adjusted their model so that it did not display this behavior.

### Anesthesia

We are of course aware that there have been many previous published reports of mitral cell recordings in the rat main olfactory bulb; many of these have not mentioned the long bursting behavior reported here as a universal characteristic of their spontaneous activity; those that have, have described this as a feature of only a proportion of mitral cells. Very few indeed have mentioned doublet spiking as a feature. In the region of the mitral cell layer, many cells fire continuously, but we were never able to confirm their identity as mitral cells by antidromic identification. Accordingly, identification of cells by recording depth or by the isopotential alone is, we believe, unreliable. As we have described here, we identified cells by stimulating the LOT below the piriform cortex – even from this distant site, the latency to antidromic spikes was just 2–3 msec. Resolving these spikes at such a short latency from both the stimulus artifact and the evoked field potential is not easy, and we noted that orthodromically activated interneurons could readily be misidentified as mitral cells because of their near‐constant latency activation.

We used urethane as an anesthetic in the studies reported here, but also made some recordings (not shown) in pentobarbitone‐anesthetized rats that were apparently indistinguishable from these. We noted that these patterns were only apparent in nasally breathing rats – in tracheotomized rats, mitral cells that we recorded were either silent or had very low spontaneous firing rates (also not shown). The use of any anesthetic is a confound of uncertain effect. The rats in these experiments were under stable, surgical levels of anesthesia throughout, and this might be expected to depress excitability (Nicoll 1972); it is not possible to be sure of the net effect, as urethane has similar, modest effects on both excitatory and inhibitory neurotransmission: recording under lighter anesthesia is not ethically acceptable, and would in any case introduce potential further confounds of pain and arousal. Rinberg et al. (2006) and Kato et al. (2012) reported that anesthesia enhances mitral cell responses to odors, apparently because the activity of inhibitory interneurons in the olfactory bulb is enhanced in wakefulness, reflecting stronger centrifugal influences on these cells.

Jiang et al. (1996) reported that 14 of 18 mitral cells that they recorded under urethane anesthesia showed long bursts similar to those described here, and mentioned that similar bursts were present using chloral hydrate as an anesthetic. When they used an inhaled anesthetic, methoxyflurane, the bursting observed depended on the depth of anesthesia, decreasing the depth of anesthesia reduced the ratio of interburst firing rate to intraburst firing rate to about 2 without affecting overall mean firing rate, and also lengthened the period of oscillations. They noted the difficulty of testing responsiveness of mitral cells to repeated stimulation when those cells exhibit marked fluctuations in firing rate, and this might indicate why this bursting appears often to have been overlooked: there may have been a common bias toward recording cells that had an apparent stable firing activity. In the present study, all antidromically identified mitral cells showed marked bursting behavior, though many of these, like the cell illustrated on the right of Figure 2A, were active during the interburst periods in the manner described by Jiang et al. (1996) under light anesthesia. However, we also recorded many cells in the same region that did not show such fluctuations, and these we were never able to activate antidromically – although some, like the cell shown in Figure 1D, were orthodromically activated to produce a spike at near constant latency that was in some cases hard to distinguish from an antidromic spike, except for a slightly longer latency combined with the jitter.

Because recordings from olfactory neurons in conscious rats have not been identified with the rigor involved in antidromic identification, there is no certainty about the discharge patterning of mitral cells in these conditions. It seems possible that the expression of bursting in mitral cells is modulated by centrifugal inhibitory input to the bulb, which is thought to be reduced under anesthesia. Jiang et al. reported no difference in overall spontaneous firing rate of the mitral cells that they recorded while varying the depth of anesthesia – hence, as this produced increased activity between bursts, it must also have produced reduced activity within bursts. In vasopressin cells of the hypothalamus, high levels of synaptic input result in long bursts (15–100 sec) separated by long silent intervals (15–40 sec), and in this case the interburst silences are the result of prolonged activity‐dependent inhibition arising from dendritic secretion of inhibitory autoregulators. Thus it seems possible that in mitral cells also, both intense bursts and profound silences are the consequence of a high sustained level of net excitatory synaptic input, and that both will be attenuated by increasing tonic inhibition.

There is little published data on the discharge patterns of identified mitral cells in the unanesthetized rat. Pager (1993) reported that the firing rate of olfactory bulb neurons, including 12 recorded from the region of the mitral cell layer as defined histologically, was “most variable, and the neuronal events could hardly be predicted from situational parameters.” Bhalla and Bower (1997) reported data from fine wire microelectrodes chronically implanted in the region of the mitral cell layer, the few interspike interval histograms that they show are all unimodal, and similar to those reported here for unimodal mitral cells but also like those of many interneurons in the region of the mitral cell layer. There is little detail given of spontaneous activity patterns, but variability consistent with long‐duration bursts is apparent in the example illustrated in their Figure 4F. Kay and Laurent (1999) recorded from cells in the region of the mitral cell layer with tungsten wire electrodes; recording sessions involved 250 successive trials (with odors and cues) at 20‐ to 60‐sec intervals, so spontaneous activity patterns over prolonged periods were not assessed; however it is appears from the records of control trials shown in their Figure 4A that there is considerable variability within cells in their spontaneous firing rate. Fuentes et al. (2008) recorded from 15 cells in the region of the mitral cell layer using implanted nichrome tetrodes during behavioral and odor‐related tasks; they reported details of refractory period, respiratory‐associated oscillations, and mean firing rate, none of which distinguish mitral cells from nonmitral cells. In all of these studies in conscious animals, technical limitations have precluded the use of antidromic identification. The mitral cell layer is just two cells thick; in our hands, recording depth, even when accompanied by electrophysiological recognition of the mitral cell layer is not a reliable guide to identifying mitral cells, and they cannot be reliably distinguished from nonmitral cells in the same region by their interspike interval distribution, mean firing rate, respiratory entrainment, or by spike size or waveform; though it is possible that the different electrode types used for conscious recordings might be more selective between cells than the class microelectrodes that we used. Nevertheless, it is likely that all the data generated in all of these studies includes at least some records from mitral cells. As conspicuous long‐duration bursting has been described by none of these, either the expression of this bursting has been disrupted by the protocols that have involved frequent trials of odors and/or odor cues, or long bursting is attenuated in the conditions of these experiments.

### Physiological significance

However, it seems unlikely to us that the intrinsic mechanisms that give rise to bursting in mitral cells are wholly artifactual consequences of anesthesia. If mitral cells have a common intrinsic ability to fire in repeated and stereotyped bursts, then these properties seem likely to be important and adaptive. Accordingly, the question that we must ask is what functional purpose might be served by such behavior.

From our studies of the responses of mitral cells to odors, it was clear that odors that elicited an excitation, when applied during a silent interval between bursts, would often trigger a prolonged burst of activity, consistent with the interpretation that bursts involve activity‐dependent regenerative mechanism. The ability to trigger prolonged bursts depended, in part, on the time elapsed since the end of the preceding burst: odor stimulation applied just after the end of a burst typically elicited only a short immediate response. The evocation of prolonged bursts may relate to previous reports of odor “afterimages” that have been recognized by calcium imaging in awake mice (Patterson et al. 2013).

However, the most obvious functional value of the burst firing can be expressed very simply, when cells are active, they can optimally detect odors that inhibit them, and when they are silent they can optimally detect odors that excite them. It seems likely that bursting intensity is modulated by the level of centrifugal input, which thereby seems likely to affect responsiveness to odors.

### Summary

We propose that bursting in mitral cells is an activity‐dependent phenomenon, on that spike activity can initiate an upstate in resting potential which, by facilitating further spiking, can result in a regenerative burst superimposed upon a plateau potential. Spike activity within bursts shows a strong tendency for spikes to recur at intervals of about 20 msec that might be partially explained by a hyperpolarizing afterpotential, but which also seems to involve a delayed depolarization that may reflect the autocrine effects of activity‐dependent dendritic glutamate release. When a sufficient level of depolarization is achieved, spiking activity commonly involves the generation of doublet spikes that we hypothesize arise as the result of a fast depolarizing afterpotential reflecting the effects of a return current following dendritic propagation of spikes. These doublets can recur, giving rise to trains of 10 or more doublets in succession, but such trains, of whatever length, are followed by an extended interspike interval, and this we hypothesize reflects the stochastic initiation of recurrent inhibition, facilitated by the spike clustering. When bursts end they are followed by an extended period in which the cell is refractory to burst initiation, indicating that the mechanism of burst termination itself may involve an activity dependent, slowly accumulating and persistent hyperpolarizing influence analogous to the influence of dendritically released dynorphin in hypothalamic vasopressin cells (Brown et al. 2013).

## Conflict of Interest

None declared.

## Supplementary Material

Figure S1

Figure S2

Figure S3

Figure S4

Figure S4A

Figure S4B

Figure S5

Figure S6

Figure S7

Figure S8

Figure S9
